# Fe^3+^ in a tetrahedral position determined the electrocatalytic properties in FeMn_2_O_4_†

**DOI:** 10.1039/d2ra04552d

**Published:** 2022-09-26

**Authors:** Caiyun Qi, Qun Liu, Yucan Dong, Guoqiang Zhang, Xingdong Jiang, Daqiang Gao

**Affiliations:** Key Laboratory for Magnetism and Magnetic Materials of MOE, Key Laboratory of Special Function Materials and Structure Design of MOE, Lanzhou University Lanzhou 730000 People's Republic of China jiangxd@lzu.edu.cn gaodq@lzu.edu.cn

## Abstract

As an electrocatalyst for the oxygen evolution reaction (OER) for water decomposition purposes, spinel ferrite materials have gained a lot of attention from many researchers. Herein, we document a co-precipitation synthesis of antitypical spinel nanoparticles (FeMn_2_O_4_) by post-annealing at different temperatures to enable modulation of the cationic oxidation state and tuning of the conversion degree for efficient and good OER performance. The electrocatalytic activity test shows that the sample annealed at 500 °C has the most optimal catalytic activity with an overpotential of 360 mV at a current density of 10 mA cm^−2^ and a Tafel slope as low as 105.32 mV dec^−1^. The formation of FeOOH during *in situ* OER promotes the catalytic activity of the catalysts. More importantly, according to the results of Brunauer–Emmett–Teller normalization, we demonstrate that the activity of the catalyst is also inseparable from the internal crystal structure. This work broadens the field of research on the electrocatalysis of spinel manganese ferrites.

## Abstract 2

Inspired by the flexibility of cation exchange and valence state variation in spinel ferrite, a high activity OER catalyst FeMn_2_O_4_ has been observed without inducing the change of composition, morphology, and atomic doping.

## Graphical abstract

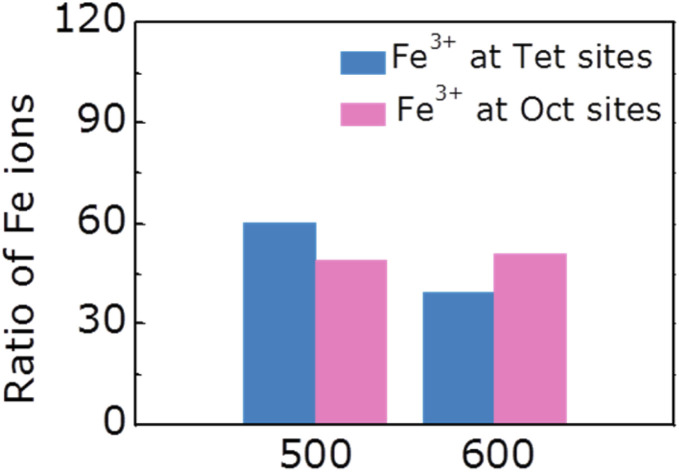

## Introduction

In recent years, with the intensification of various terrible phenomena that threaten the survival of mankind, such as the global energy crisis, environmental crisis, and even the scientific and technological crisis, it is indispensable to seek a new energy system that is clean, efficient and sustainable.^1,2^ In the future, ways to create, store and use renewable energy are considered efficient, and electrolysis of water to produce hydrogen is one such technology.^3^ Oxygen evolution is crucial in the process of water decomposition and carbon dioxide reduction. However, due to the inherent kinetic sluggishness of a series of proton-coupled electron transfer steps, significant challenges remain in the search for robust and cost-effective OER electrocatalysts.^4^ The catalytic activity of noble-metal-based electrocatalysts such as IrO_2_ and RuO_2_ has been extensively studied, and they possess outstanding water oxidation activity and stability in both acidic and alkaline solutions, but there is no doubt that the abundance and high cost of Earth's needs block their market applications.^5,6^ Therefore, exploring promising alternative electrocatalysts is of great interest to the community.

Despite effective tailoring of noble metals and alloys to allow important electrochemical processes, developing resourceful and less costly materials remains a significant problem. Spinel oxides have piqued the scientific imagination and found practical applications in a variety of sectors, including spintronic devices, data storage, supercapacitors, biomedicine, light absorption, environmental remediation, and so on.^7–13^ One of the causes for spinel oxides' diverse physicochemical features is their structure, which has the generic chemical formula AB_2_O_4_ (where A and B are metal ions). Spinels are classified into three forms based on the ion distribution between the tetrahedral A and octahedral B sites: normal, inverse, and mixed spinels.^14^ The distribution of cations is affected by the preparation conditions of spinel oxides, such as annealing time, annealing temperature, atmosphere pressure and cooling rate.^15–18^ Mn-based spinels such as CoMn_2_O_4_, CuMn_2_O_4_ and NiMn_2_O_4_ were studied as OER catalysts.^19–26^ It has been reported that MnFe_2_O_4_, one of the Fe–Mn spinels, is a stable OER catalyst. In such a magnetic ferrite, Mn ions mostly occupy A sites and Fe ions mainly occupy B sites.^27–30^ However, the pure phase MnFe_2_O_4_ requires an overpotential of about 430 mV to reach a current density of 10 mA cm^−2^. As an OER catalyst, the catalytic activity of MnFe_2_O_4_ is obviously not excellent. It is worth noting that the effect of different positions occupied by cations on the catalytic activity of spinel is still unclear and needs to be further explored.

Inspired by the flexibility of cation exchange and valence state variation in spinel ferrite, a high activity OER catalyst FeMn_2_O_4_ has been observed without inducing the change of composition, morphology, and atomic doping. Our experimental results show that the sample has the best OER catalytic activity when the annealing temperature is 500 °C, in which the overpotential is only 360 mV at the current density of 10 mA cm^−2^ and the slope of Tafel is 105.32 mV dec^−1^. Further characterization of the sample after the OER tests reveals the presence of the key substance of amorphous FeOOH, which promotes electrocatalytic activity. We demonstrate that the active site in the Mn-based spinel system is the tetrahedral Fe^3+^, and the more ions in the system, the better catalytic activity. Our findings broaden the field of research on the electrocatalysis of spinel manganese ferrites.

## Experimental part

### Preparation of spinel oxides

As shown in Fig. S1,† FeCl_3_·6H_2_O and MnCl_2_·4H_2_O powders were dissolved in water to form solutions with concentrations of 0.8 M and 0.4 M respectively. Then slowly pour the above two solutions into 6 M sodium hydroxide solution. Next, the slurry was placed in a boiling water bath for 2 hours. The digested slurry is washed several times with deionized water. Finally, the obtained particles were dried in air at 145 °C for 2 hours to extract the precursor. The precursor is placed in a tubular furnace, heated at 300 °C, 400 °C, 500 °C and 600 °C respectively at a heating rate of 10 min °C^−1^ for 3 hours, and then cooled in the furnace to obtain the target sample. Here we named the resulting samples as 300, 400, 500 and 600, respectively.

### Materials characterizations

The phase of FeMn_2_O_4_ was characterized by an X-ray diffraction analyzer (XRD) with Cu Kα radiation. The crystal structure of the samples was studied by Raman spectroscopy (LabRAM HR Revolution) with wavelength of 532 nm and wave number of 200–1000 cm^−1^. Scanning and transmission electron microscopy (SEM/TEM) images were carried out on Hitachi S-4800 and Tecnai G2 F30, FEI, respectively. The elemental valence analysis and occupation state of Fe in the spinel oxides were evaluated using X-ray photoelectron spectroscopy (XPS, Kratos Axis Ultra), and Mössbauer spectroscopy, respectively. Brunauer–Emmett–Teller (BET) measured with a Micromeritics ASAP 2010 system. The magnetism was measured by a vibrating sample magnetometer (VSM, EV9, MicroSense).

### Electrochemical measurements

The preparation of the electrolyte is described as follows. Firstly, weigh 10 mg of sample and equal mass toner dissolved in about 40 mL of solvent petroleum ether for about 3 hours so that they are fully mixed and evenly dispersed. Then, the mixture was centrifuged to pour out the supernatant, and the remaining precipitate is placed in a blast drying box at 60 °C to dry to obtain a mixed powder. Then 6 mg of the above powder was dissolved in a mixture of 1470 μL dimethylformamide and 30 μL Nafion glue and ultrasonically obtained electrolyte for about 3 hours. Finally, 3 μL of electrolyte droplets were dried on a glassy carbon electrode using a pipette and then tested in 1 M KOH solution using a three-electrode system after three repetitions. The potential calculation formula is: *E*_(RHE)_ = *E*_Ag/AgCl_ + 0.197 + 0.059 × pH, where RHE is a reversible hydrogen electrode.

## Results and discussion

**Fig. 1:**
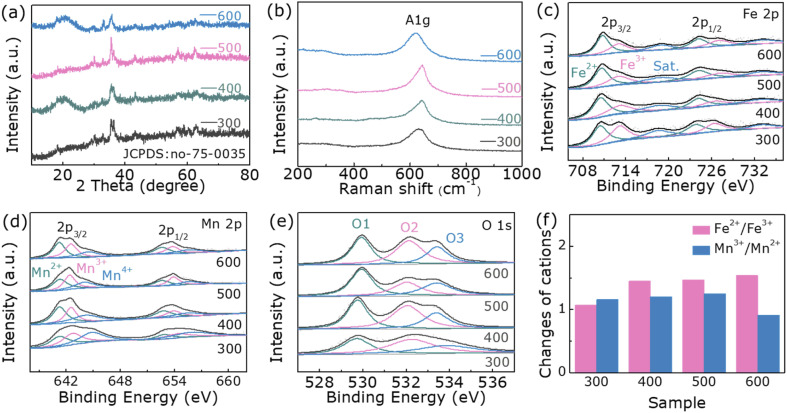
(a) X-ray diffraction patterns, (b) Raman spectrum, (c) Fe 2p spectrum, (d) Mn 2p spectrum and (e) O 1s spectrum of sample 300, 400, 500 and 600. (f) Relative strength of different valence cations.

The diffraction peaks of samples after annealing in air at 300, 400, 500, and 600 °C, respectively, correspond to those of spinel FeMn_2_O_4_ (JCPDS card No: 75-0035) (Fig. 1a). The 2*θ* values of the diffraction peaks at 18.04°, 29.67°, 34.94°, 36.54°, 42.45°, 56.11° and 61.59° correspond to the (111), (220), (311), (222), (400), (511) and (440) planes, respectively, verified the formation of the FeMn_2_O_4_ phase. The Raman spectra measured ranging from 200 to 1000 cm^−1^ are depicted in Fig. 1b. Normally, five Raman active modes including A_1g_, E_1g_, and 3 T_2g_ are detected for FeM_2_O_4_ (M = divalent metal ion) by factor group analysis.^31^ The peaks detected in our samples at 640 cm^−1^ in the Raman spectra are assigned to A_1g_ and this is the result of symmetrical stretching of Fe–O bonds at tetrahedral positions.^31^ The ultra-low frequency mode attributed to the octahedral site is too weak to be very visible. Unrelated patterns are not detected. This corresponds to XRD data.

To further explore the effect of temperature on the cation valence states, X-ray photoelectron spectroscopy (XPS) was applied to characterize samples. Detailed observations of the oxidation states of the elements are obtained from the Fe 2p, Mn 2p and O 1s high-resolution spectra (Fig. S3†). As shown in Fig. 1c, the Fe 2p spectrum of FeMn_2_O_4_ can be matched with two major characteristic peaks and satellite peaks. The peaks located at 713.1 eV and 727.0 eV are assigned to Fe^3+^, and the peaks at 710.5 eV and 724.2 eV indicate the existence of Fe^2+ 24^ (Table S1, ESI†). In Fig. 1d, the fitted peaks at 641.2 eV and 652.6 eV correspond to Mn^2+^, the peaks at 645.6 eV and 653.9 eV are attributed to Mn^3+^, and peaks at 644.5 eV and 655.6 eV are assigned to Mn^4+^ caused by slight oxidation of the surface.^32^ (Table S2†). As indicated in Fig. 1e, the O 1s spectrum composed of O_1_, O_2_, and O_3_ represent the metal–oxygen bond, the number of oxygen defect sites at the surface and the absorbed H_2_O at the surface, respectively.^32^ (Table S3†). To observe the effect of temperature on the cation valence states of samples more intuitively, we list the relative strengths of the different valence states of Fe and Mn cations in Fig. 1f. As expected, the temperature cause a change in the internal structure of the spinel FeMn_2_O_4_, transforming the sample to its ideal state. As the post-annealing temperature increases, Fe^2+^/Fe^3+^ increases while Mn^3+^/Mn^2+^ increases slightly, which is favorable for good OER performance. It is not difficult to see that when the temperature is higher than 500 °C, Mn^3+^/Mn^2+^ does not increase but falls. This is related to the degree of inversion of the spinel.

**Fig. 2:**
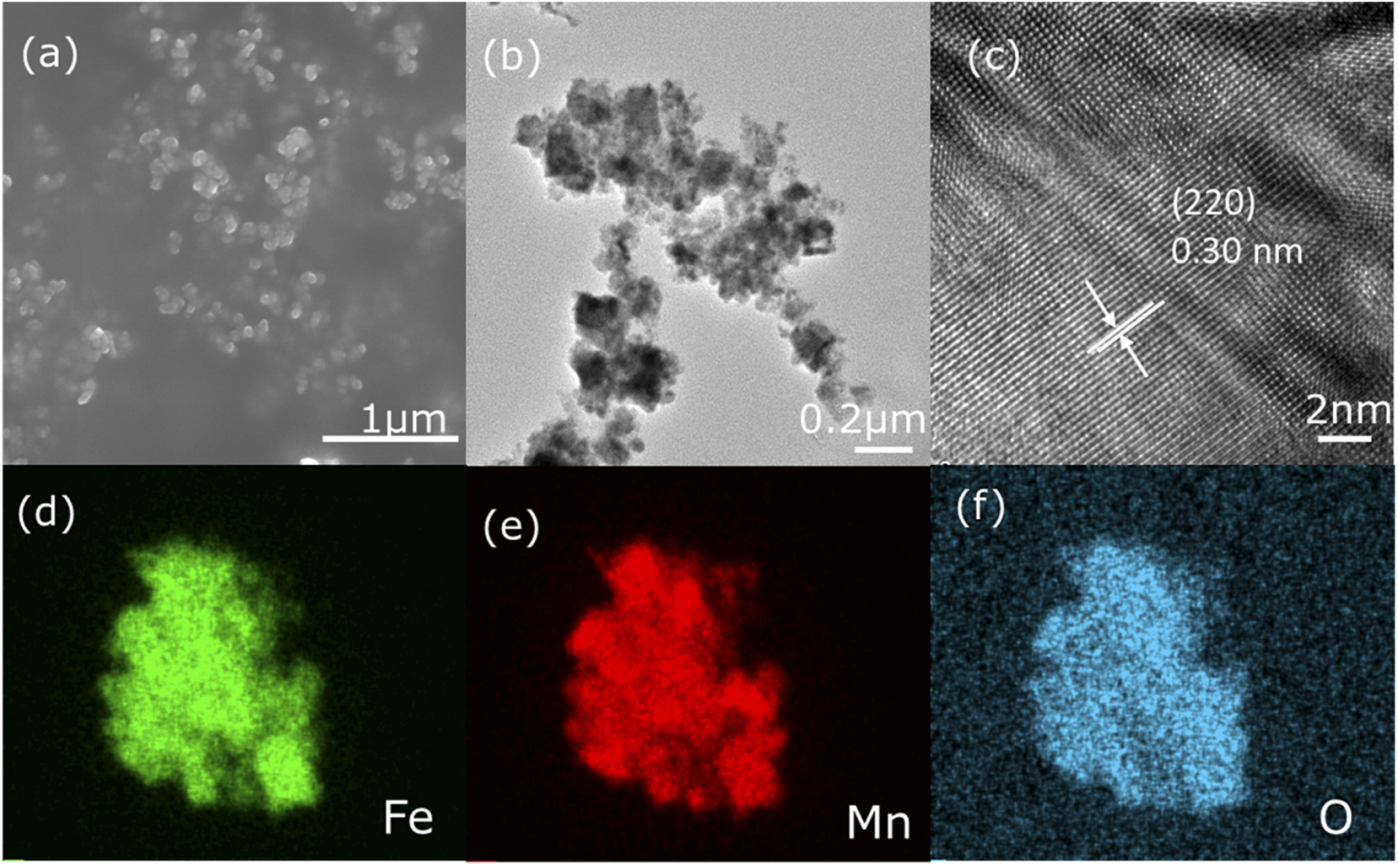
(a) SEM picture, (b) TEM graphics, and (c) HRTEM image of sample 500. (d–f) Elemental mapping images of sample 500.

The structure and morphology of sample 500 are considered using SEM and TEM. The results are shown in Fig. 2a and b, where the sample exhibits a spherical shape formed by the aggregation of nanoparticles. The fringe spacing of 0.30 nm corresponding to the FeMn_2_O_4_ crystal plane (220) is prominently observed in the HRTEM image in Fig. 2c and this echoes the XRD results. In addition, Fig. 2d–f displays the element mapping images of sample 500, and results verify that Fe, Mn, and O are uniformly scattered in the sample.

**Fig. 3:**
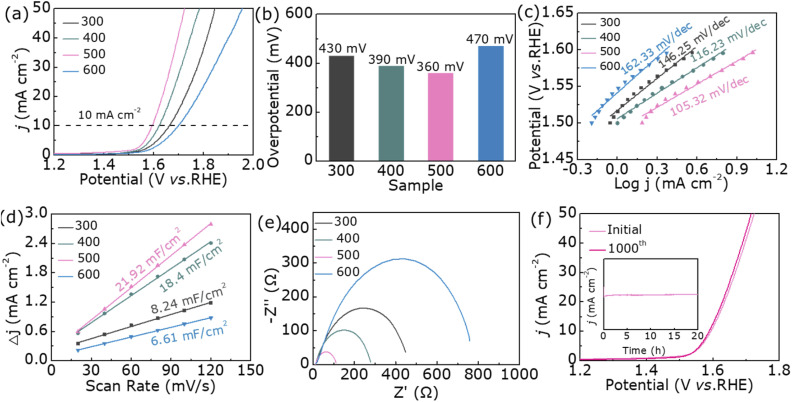
(a) OER LSV curves, (b) *η*10 and (c) Tafel plots of 300, 400, 500, and 600 for the OER. (d) Double-layer capacitance plots (*C*_dl_) and (e) Nyquist plots of 300, 400, 500, and 600. (f) LSV curves of the sample 500 catalyst before and after 1000 cycles; inset is its chronoamperometry test.

In Fig. 3a, the LSV curves shows the OER catalytic activity of samples 300, 400, 500, and 600. With the increase of the annealing temperature, the OER activity of the sample shows a significant difference, of which sample 500 shows the best OER catalytic performance while sample 600 has the largest onset potential. The *η*_10_ summarized from Fig. 3a is shown in Fig. 3b. The best-notable sample 500 expresses 360 mV (*η*_10_). Besides, the cyclic voltammetry curves (Fig. S2†) displays that *C*_dl_ (Fig. 3d) of sample 500 is about 21.92 mF cm^−2^, higher than that of 300 (8.24 mF cm^−2^), 400 (18.4 mF cm^−2^) and 600 (6.61 mF cm^−2^). Fig. 3c shows the Tafel plots calculated from Fig. 3a. We can found that sample 500 holds the lowest Tafel slopes (105.32 mV dec^−1^), displaying the supreme kinetic activity. In addition, we adopt the electrochemical impedance spectroscopy (EIS) in Fig. 3e to represent the charge transfer resistance. As shown, sample 500 has the smallest arch, indicating that it has the best OER activity and fastest charge transfer capability. The OER performance comparison before and after 1000 cycle optimization is shown in the Fig. 3f, where the *η*_10_ shows hardly difference. In addition, as can be seen from the inset of Fig. 3f, the current density of sample 500 remains almost unchanged over 20 hours of continuous electrolysis, which reflects its excellent stability during the OER catalysis process.

**Fig. 4:**
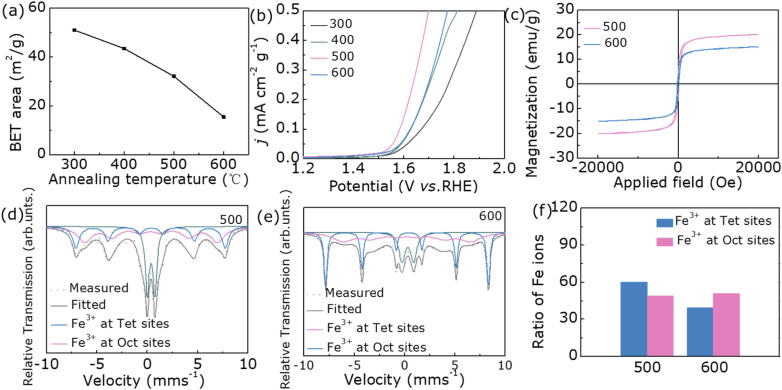
(a) BET surface area of all samples, (b) BET surface area normalized LSV plot, (c) magnetic hysteresis loops, (d) Mössbauer spectra of 500, and (e) 600 at room temperature. (f) The ratio of Fe ions in different sites for 500 and 600.

In general, good OER catalytic activity is inseparable from the number of active sites on the catalyst surface, so all samples are characterized by their BET results.^33,34^ As shown in Fig. 4a, as the annealing temperature rises, the specific surface area gradually decreases. From the BET normalized LSV plots in Fig. 4b, the OER performance of sample 500 is still the best, but sample 600 is no longer the worst. This result shows that the OER catalytic activity of the samples of this system depends not on the number of active sites but also on its crystal structure. Based on the current experimental results, we select sample 500 and sample 600 as representatives to further investigate the effect of cation occupancy on OER performance. At the beginning, we studied the magnetic properties of two samples. And the hysteresis loops of samples 500 and 600 were characterized by vibrating sample magnetometer (VSM) (Fig. 4c). As can be seen from the VSM data, the saturated magnetization for sample 500 (20.2 emu g^−1^) is larger than that of sample 600 (15.1 emu g^−1^). By consulting the literature, we found that the magnetic and cationic occupancy changes of the samples are correlated.^35–37^ Therefore, we further used the Mössbauer spectrum to study the occupancy sites of Fe ions.

In order to explore whether the structural changes in nanoparticles are caused by Fe^3+^, the Mössbauer spectra measured at room temperature are shown in Fig. 4d and e, and Table S4† lists the fitted hyperfine parameters. The spectrum can be fitted by two hexahedrons composed of Fe ions occupying tetrahedron and octahedron. For the Fe^3+^ ion in sample 500, it occupies tetrahedron to form a sextet with a smaller hyperfine magnetic field of 41.0 T, while it occupies octahedron with a hyperfine magnetic field of 46.1 T. Meanwhile, Fe^3+^ ions in another sample occupy tetrahedron to form FeMn_2_O_4_ sextets with it of 50.2 T, and Fe^3+^ ions in octahedrons with it of 39.4 T. The presence of Fe^3+^ ions in both positions shows that samples 500 and 600 are hybrid spinels (Fe_0.60_Mn_0.40_)_T_(Fe_0.40_Mn_1.60_)_O_O_4_ and (Fe_0.49_Mn_0.51_)_T_(Fe_0.51_Mn_1.49_)_O_O_4_, respectively (Fig. 4f).^26^ The characterization of the Mössbauer spectroscopy further demonstrates that the internal crystal structure of both samples is different. The more Fe^3+^ in the tetrahedral position, the stronger magnetism and the more beneficial to OER performance.

**Fig. 5:**
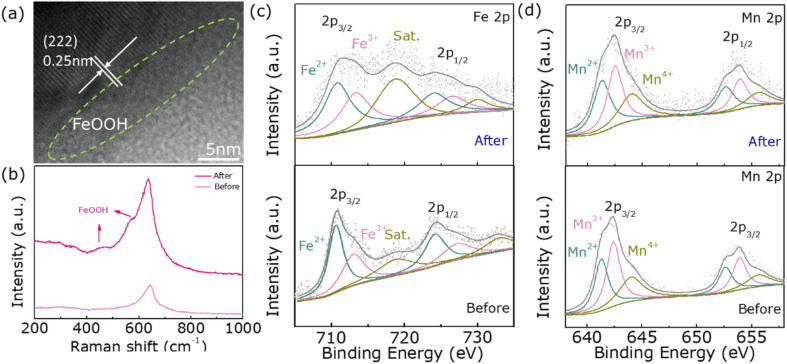
(a) HRTEM image of 500 after the OER durability test, (b) Raman spectrum, (c) Fe 2p spectrum, and (d) Mn 2p spectrum before and after the OER test.

Since the OER test is performed in a strong alkaline solution, there is also the effect of high potential and high current density, which may lead to structural and chemical changes in the electrocatalyst. Moreover, in the existing studies, it has been proposed that the surface reconstruction of the catalyst is an important reason for the performance improvement so we have characterized sample 500 after catalysis.^38^Fig. 5a is a high-resolution plot of sample 500 after a long durability test, which shows that an amorphous layer with a thickness of about a few nanometers was produced on the surface of the catalyst. This may be attributed to the formation of FeOOH due to the elevated oxidation state of Fe ions. To confirm this conjecture, the sample is subsequently tested by Raman, and the peaks corresponding to FeOOH do appear around 580 cm^−1^ and 470 cm^−1^ in Fig. 5b.^39,40^ In addition, to further and more fully demonstrate the increase in the oxidation state of Fe ions, XPS characterization is also used. Fig. 4c shows that the proportion of Fe^3+^ after OER increases significantly and the valence state of Mn does not change significantly. Meanwhile, O_3_ in Fig. S4† decreases a lot, which is the consequence of its promoting oxidation. The above several characterizations confirm the formation of FeOOH in the post-OER amorphous layer, which is the essential reason for the increase in electrocatalytic activity.

## Conclusions

In summary, a series of FeMn_2_O_4_ nanoparticles are prepared by co-precipitation and annealed at different temperatures. The results show that the material obtained at 500 °C exhibits the best OER activity under alkaline conditions, requiring only a low overpotential of 360 mV to drive a current density of 10 mA cm^−2^. We use XRD, XPS, Mössbauer spectroscopy and other characterization methods to analyze the phase structure, ion valence information and Fe ion occupancy to verify the best performance of the sample. This discovery opens a new door for OER research in the spinel family.

## Data availability

The data that supports the findings of this study are available within the article (and its ESI†).

## Conflicts of interest

There are no conflicts to declare.

## Supplementary Material

RA-012-D2RA04552D-s001
