# Supplementary material for: Fe3+ in a tetrahedral position determined the electrocatalytic properties in FeMn2O4
Source: RSC Adv. 2022 Sep 26;12(42):27206–11. doi: 10.1039/d2ra04552d (PMC9510904; doi:10.1039/d2ra04552d)
Supplement: RA-012-D2RA04552D-s001 [file RA-012-D2RA04552D-s001.pdf]

Electronic Supplementary Material (ESI) for RSC Advances.
This journal is © The Royal Society of Chemistry 2022

Supporting Information

Fe^{3+} in Tetrahedral Position Determined Electrocatalytic Properties in $FeMn_2O_4$

Caiyun Qi, Qun Liu, Yucan Dong, Guoqiang Zhang, Xingdong Jiang*, and Daqiang Gao*

Key Laboratory for Magnetism and Magnetic Materials of MOE, Key Laboratory of Special Function Materials and Structure Design of MOE, Lanzhou University, Lanzhou 730000, People's Republic of China.

*Corresponding author. E-mail: jiangxd@lzu.edu.cn, gaodq@lzu.edu.cn

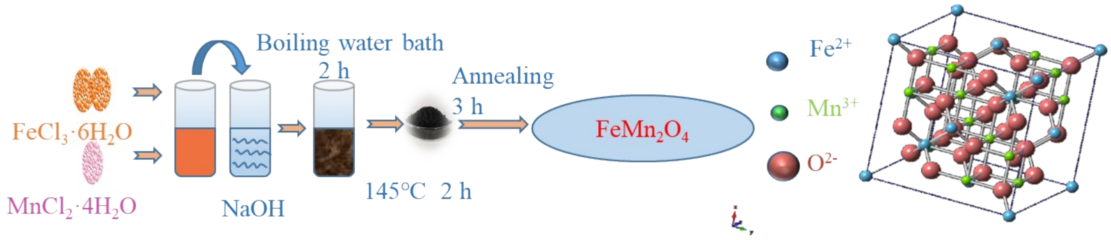

Fig. S1 Schematic illustration of $FeMn_2O_4$.

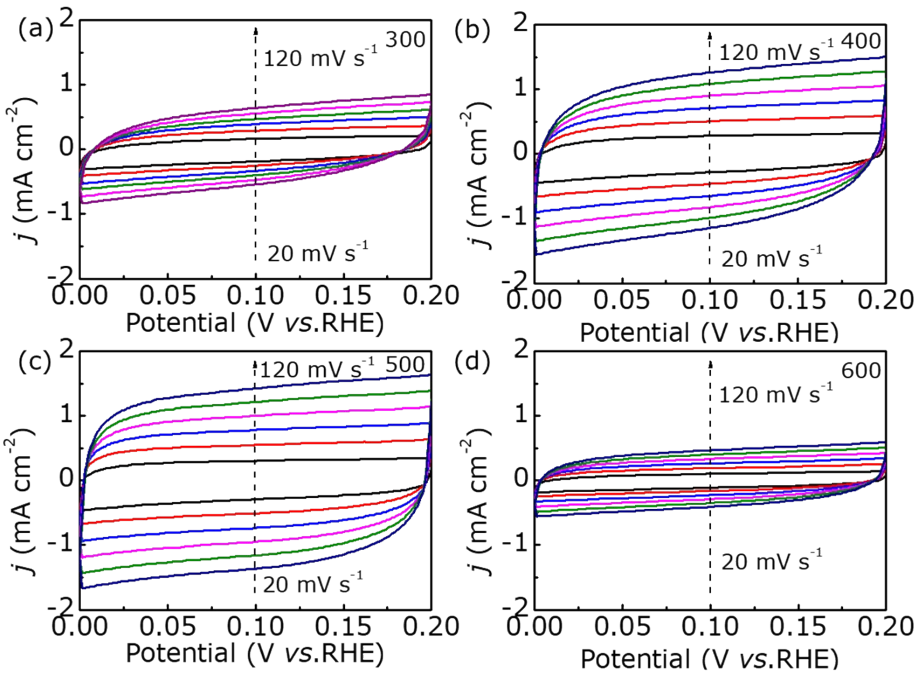

Fig. S2 CV curves of sample(a) 300, (b) 400, (c) 500 and (d) 600.

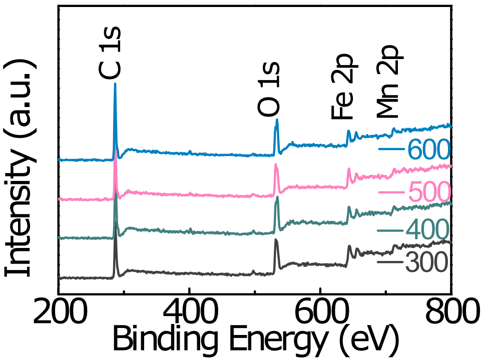

Fig. S3 XPS survey spectrum of the sample 300, 400, 500 and 600

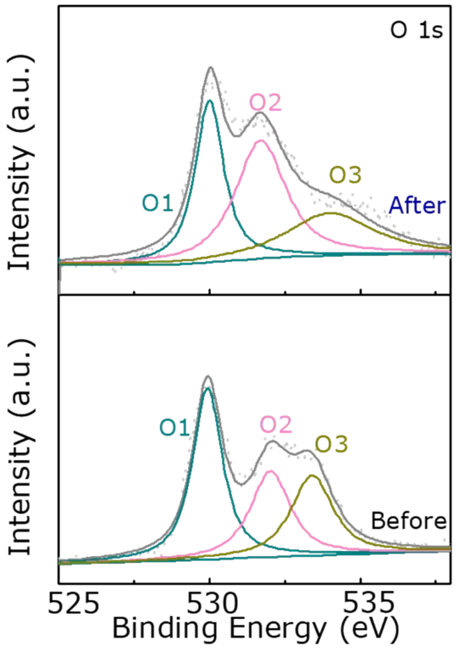

Fig. S4 O 1s spectrum before and after the OER test.

Table S1. Position of Fe 2p component of $FeMn_2O_4$ nanoparticles.

Peak	Species	B.E.(eV)	Area
300-Fe2p$_{3/2}$	Fe^{2+}	710.42	1200.3
	Fe^{3+}	713.16	1118.28
400-Fe2p$_{3/2}$	Fe^{2+}	710.48	1259.07
	Fe^{3+}	713.18	852.25
500-Fe2p$_{3/2}$	Fe^{2+}	710.62	1148.56
	Fe^{3+}	713.11	794.4
600-Fe2p$_{3/2}$	Fe^{2+}	710.78	1004.26
	Fe^{3+}	713.02	648.91
300-Fe2p$_{1/2}$	Fe^{2+}	723.77	713.22
	Fe^{3+}	726.38	640.72

400-$Fe2p_{1/2}$	Fe^{2+}	724.14	628.76
	Fe^{3+}	726.39	436.44
500-$Fe2p_{1/2}$	Fe^{2+}	724.22	762.86
	Fe^{3+}	727.19	554.83
600-$Fe2p_{1/2}$	Fe^{2+}	724.21	596.29
	Fe^{3+}	724.04	502.23

Table S2. Position of Mn 2p component of $FeMn_2O_4$ nanoparticles.

Peak	Species	B.E.(eV)	Area
300-$Mn2p_{3/2}$	Mn^{2+}	641.24	1582.91
	Mn^{3+}	642.69	1834.54
	Mn^{4+}	644.82	1980.02
400-$Mn2p_{3/2}$	Mn^{2+}	641.28	1944.22
	Mn^{3+}	642.54	1448.6
	Mn^{4+}	644.2	1513.82
500-$Mn2p_{3/2}$	Mn^{2+}	641.29	1202.34
	Mn^{3+}	642.38	1512.2
	Mn^{4+}	644.18	982.13
600-$Mn2p_{3/2}$	Mn^{2+}	641.24	1574.62
	Mn^{3+}	642.55	1436.96
	Mn^{4+}	644.45	946.68
300-$Mn2p_{1/2}$	Mn^{2+}	652.85	616.66
	Mn^{3+}	654.35	885.8
	Mn^{4+}	655.89	1485.14
400-$Mn2p_{1/2}$	Mn^{2+}	652.79	998.58
	Mn^{3+}	653.92	813.66
	Mn^{4+}	655.51	800.28
500-$Mn2p_{1/2}$	Mn^{2+}	652.61	612.13
	Mn^{3+}	653.9	752.14
	Mn^{4+}	655.62	451.24
600-$Mn2p_{1/2}$	Mn^{2+}	652.62	865.98
	Mn^{3+}	653.81	803.69
	Mn^{4+}	655.64	658.36

Table S3. Position of O 1s component of $FeMn_2O_4$ nanoparticles.

Peak	B.E.(eV)	Area
300-O1	529.72	2999.72
300-O2	532.25	4447.02
300-O3	533.84	2988.86
400-O1	529.75	3971.91

400-O2	532.05	4848.13
400-O3	533.39	2725.98
500-O1	529.93	3941.03
500-O2	532.03	2620.55
500-O3	533.4	2563.84
600-O1	529.92	3703.94
600-O2	532.13	4352.68
600-O3	533.43	2517.76

Table S4. The Mössbauer Parameters: Isomer Shift (IS), Quadrupole Splitting (QS), Average Magnetic Hyperfine Field (H), Width, and Relative Area (Area) Obtained by Spectral Fitting.

Samples	Subspectrum	IS(mm/s)	QS(mm/s)	H(T)	Width(mm/s)	Area(%)
500	D1	0.37	0.76		0.47	20.9
	D2	0.35	-7.69		2.33	13.3
	D3	0.37	1.58		0.77	10.2
	M1	0.35	0.06	41.03	1.62	33.5（60.4)
	M2	0.36	-0.07	46.09	0.76	22.0（39.6)
600	D1	0.31	1.15		0.7	15.8
	D3	0.27	6.84		1.77	22.1
	M1	0.33	-0.2	50.26	0.35	30.5（49.0)
	M2	0.23	-0.06	39.46	2.36	31.7（51.0)

Table S5. Comparison of OER catalytic parameters in this work with other catalysts.

Catalysts	Electrode potential (j=10 mA cm^{-2})	Electrolyte	References
Mn_2O_3	580 mV	0.1 M KOH	1
Mn_3O_4	323 mV	1 M KOH	2
MnO_2	570 mV	0.1 M KOH	3

$CoMn_2O_4$	310 mV	0.1 M KOH	4
$NiFe_2O_4$	381 mV	1 M KOH	5
R-Fe_3O_4	320 mV	1 M KOH	6
$FeCo_2O_4$	393 mV	1 M KOH	7
$FeMn_2O_4$	360 mV	1 M KOH	This work

References

1. K.L. Pickrahn, S.W. Park, Y. Gorlin, H.B.R. Lee, T.F. Jaramillo, S.F. Bent, *Adv. Energy. Mater.*, 2012, **2**, 1269-1277.

2. P.C. Nagajyothi, R. Ramaraghavulu, K. Munirathnam, K. Yoo, J. Shim, *Int. J. Hydrogen. Energ.*, 2021, **46**, 13946-13951.

3. J. He, M. Wang, W. Wang, R. Miao, W. Zhong, S.Y. Chen, S. Poges, T. Jafari, W. Song, J. Liu, S.L. Suib, Hierarchical Mesoporous NiO/MnO_2@PANI Core-Shell Microspheres, *ACS. Appl. Mater. Inter.,* 2017, **9**, 42676-42687.

4. A. Bahadur, W. Hussain, S. Iqbal, F. Ullah, M. Shoaib, G. Liu, K. Feng, *J. Mater. Chem. A.*, 2021, **9**, 12255-12264.

5. V. Maruthapandian, M. Mathankumar, V. Saraswathy, B. Subramanian, S. Muralidharan, *ACS. Appl. Mater. Inter.*, 2017, **9**, 13132-13141.

6. X. Y. Zhang, B. Y. Guo, Z.Y. Lin, B. Dong, Q.W. Chen, Y.W. Dong, M. Yang, L. Wang, C.G. Liu, Y.M. Chai, *Int. J. Hydrogen. Energ.*, 2020, **45**, 15476-15482.

7. A. Kundu, A.I. Robby, A. Shit, H.J. Jo, S.Y. Park, *Mater. (Basel)*, 2020, **13**, 3119.
